# Pain and Disgust: The Facial Signaling of Two Aversive Bodily Experiences

**DOI:** 10.1371/journal.pone.0083277

**Published:** 2013-12-09

**Authors:** Miriam Kunz, Jessica Peter, Sonja Huster, Stefan Lautenbacher

**Affiliations:** 1 Physiological Psychology, Otto-Friedrich University Bamberg, Bamberg, Germany; 2 Department of Neurology, University Medical Center Freiburg, Freiburg, Germany; The University of Queensland, Australia

## Abstract

The experience of pain and disgust share many similarities, given that both are aversive experiences resulting from bodily threat and leading to defensive reactions. The aim of the present study was to investigate whether facial expressions are distinct enough to encode the specific quality of pain and disgust or whether they just encode the similar negative valence and arousal level of both states. In sixty participants pain and disgust were induced by heat stimuli and pictures, respectively. Facial responses (Facial Action Coding System) as well as subjective responses were assessed. Our main findings were that nearly the same single facial actions were elicited during pain and disgust experiences. However, these single facial actions were displayed with different strength and were differently combined depending on whether pain or disgust was experienced. Whereas pain was mostly encoded by contraction of the muscles surrounding the eyes (by itself or in combination with contraction of the eyebrows); disgust was mainly accompanied by contraction of the eyebrows and—in contrast to pain—by raising of the upper lip as well as the combination of upper lip raise and eyebrow contraction. Our data clearly suggests that facial expressions seem to be distinct enough to encode not only the general valence and arousal associated with these two bodily aversive experiences, namely pain and disgust, but also the specific origin of the threat to the body. This implies that the differential decoding of these two states by an observer is possible without additional verbal or contextual information, which is of special interest for clinical practice, given that raising awareness in observers about these distinct differences could help to improve the detection of pain in patients who are not able to provide a self-report of pain (e.g., patients with dementia).

## Introduction

Facial expressions are critical elements in nonverbal communication, which allow observers to infer the internal state of others and - in case of negative states - to be alarmed of impeding danger or be prepared for empathic behavior [1–3]. The question is how specific this warning signal can be. Is the observer informed about an impeding threat in general by suggesting that somebody is experiencing a negative affective state or can facial expressions point to the specific type of threat? Clearly, facial expressions can only be specifically perceived if they are sufficiently distinct. 

Pain and disgust are suitable models to address this question because of their minimal expressive difference [4,5], which allow for very critical testing. Both states are elicited by harmful or potentially harmful stimuli (that can be threatening to our physical integrity) and are characterized by strong feelings of unpleasantness and both states result in defensive behavior [6–8]. However, despite of these similarities, the respective threat to the body and the resulting subjective experiences are fundamentally different. Are facial expressions during the experience of pain and disgust specific enough to capture the distinctness of these two negative affective states? When looking at previous findings, there is on the one hand evidence that would suggest high distinctness of facial expressions of pain and disgust whereas on the other hand some findings favor the assumption of expressive overlap between the two facial expressions. 

Evidence for the latter can be found in research that focused on the facial muscle movements elicited during pain and disgust. Using the Facial Action Coding System (FACS [9]) – which is considered to be the gold standard in facial expression research – it has been shown that the experience of disgust elicits contraction of the eyebrows (Action Unit (AU) 4), nose wrinkling (AU9), upper lip raise (AU10), jaw drop (AU25/26/27), raising of the chin (AU17) and narrowing the eyes (AU6/7) [10–12]. Amongst these facial movements, the activity of the musculus levator labii superior (which leads to the upper lip raise and nose wrinkling) seems to be the most central one whereas for the other movements there is no complete consensus between studies. Interestingly, pain similarly elicits contraction of the eyebrows (AU4), nose wrinkling (AU9), upper lip raise (AU10) and narrowing the eyes (AU6/7). In addition, closing the eyes for more than 0.5 seconds (AU43) has also been shown to occur in the context of pain [13–15]. Thus, there seems to be a great overlap between the facial movements that are elicited during pain and disgust, which might challenge the idea of distinctness between the two facial expressions. In line with this, observers often confuse facial expressions of pain with those of disgust [4,5,16,17] which might not be surprising given the similarity in facial ovements involved in the two expressions. 

However, a certain degree of expressive overlap or confusion of both states by observers would not necessarily preclude sufficient distinctness of facial expressions to act as specific warning signals. Especially since observers can distinguish facial expressions of pain and disgust clearly above chance level, there is some evidence for sufficient distinctness of these two facial expressions. Furthermore, the overlap might be in fact less than it seems to be due to the characteristics of the material used for disgust induction. Disgust has often been induced using pictures and films that also contain pain-related content, i.e. individuals with injuries or pictures of ripped off limbs, mutilations, etc. [18,19]. Hence, it is difficult to decide whether facial expressions elicited by these confounded stimuli are specific for disgust or are also representative for pain. 

So far, the overlap in facial expressions between pain and disgust has not yet been systematically studied. The study would be worthwhile just because of the proximity of the two facial expressions and the risk of overlap. If the facial expressions are distinct in these two cases they are in other cases too.

No study has yet investigated these two facial expressions in one sample; which is the only way to test whether these two facial expressions are – despite similarities – still distinct in the same person (intra-individual comparison). Thus, the aim of the present study was to investigate whether individuals facially encode the specific emotional quality of pain and disgust (leading to distinct facial expressions) or whether they simply encode the similar negative valence and arousal of both states (leading to similar facial expressions). We assessed facial and subjective responses to pain (induced by heat stimuli) and to disgust (induced by pictures) in one sample and used disgust stimuli with and without pain-related content to be able to compare facial expressions elicited by “pure” disgust and by disgust due to pain-confounded contents. Based on previous findings, we hypothesized that pain and disgust elicit a very similar set of single facial muscle movements. However, given that observers can differentiate between pain and disgust expressions above chance level, we expected that the distinctness between the two facial expressions might rely on different combinations of these single facial movements. 

## Methods

### Participants

Sixty healthy volunteers (30 males and 30 females, mean age 22.9 ± 4.3), were recruited via advertisements posted in the university buildings of the University of Bamberg. Exclusion criteria were current experience of acute or chronic pain, psychological illnesses (especially any kind of anxiety disorders) and physical illnesses. None of the participants had taken analgesics, psychotropic medication or alcohol the day before testing. 

#### Ethics statement

All participants provided written informed consent and received monetary compensation (25 €) or course credits for their participation. The study (including the consent procedure) was approved by the ethics committee of the University of Bamberg. 

### Materials and procedure

#### General protocol

**Figure 1:**
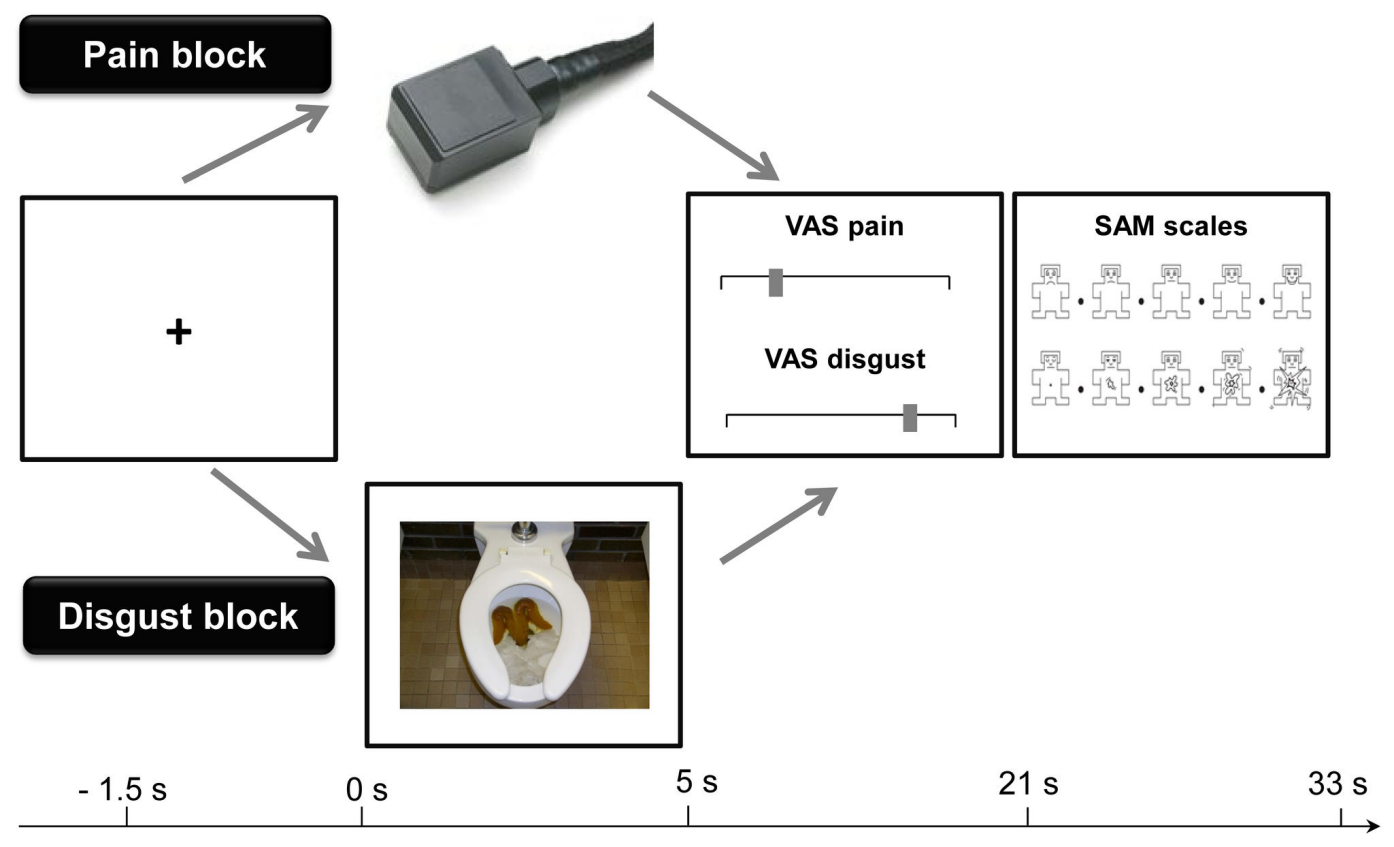
Experimental procedure. Following a fixation cross, participants received a 5 second heat stimulus (painful and non-painful heat, pain block) or viewed for 5 seconds a picture (of neutral, pain-disgust or “pure” disgust content, disgust block). Participants rated pain, disgust as well as valence and arousal after each stimulus.

The study was composed of two experimental blocks: a pain induction block and a disgust induction block (see Figure 1). The order of blocks was balanced across participants, with half of the participants starting with the pain induction block, whereas the other half started with the disgust induction block. 

In the pain block participants received heat stimulation of non-painful and painful heat intensities. In the disgust block participants viewed pictures of different emotional content: neutral (as fillers), positive (to counteract an unspecific lowering of mood), disgust due to pain-related content (e.g. mutilation) and “pure” disgust pictures (e.g. bodily excrements). In both the pain and the disgust blocks participants were seated in front of a 19-inch computer screen positioned 50 cm in front of them. Each target stimulus (heat or picture stimuli) was preceded by a fixation cross (duration 1s - 3s) to orient eye gaze to the center of the screen and to minimize eye and head movements for later off-line analyses of facial display (see Figure 1). The fixation cross was followed by 5 s of heat stimulation (pain block) or by 5 s of picture presentation (disgust block), respectively. After stimulus offset participants had to rate their experiences on separate scales (see Figure 1). To familiarize participants with the rating procedures, two familiarization trials were conducted at the beginning of each block. During the whole session, which lasted almost 2 hours, participants sat upright in a comfortable chair. 

#### Pain block

Following a previous protocol that has been shown to successfully elicit facial expressions of pain [20,21], pain was induced by use of a Peltier-based, computerized thermal stimulator (Medoc TSA-2001; Medoc Ltd, Ramat Yishai, Israel) with a 3 × 3 cm^2^ contact probe attached to the outer part of the left lower leg (midpoint between ankle and knee). To ensure that painful stimuli were indeed perceived as being painful without exceeding individuals pain tolerance, we adjusted stimulation temperature to the individual pain threshold. Thus, heat pain thresholds were determined first, using the method of adjustment. Participants were asked to adjust pain threshold starting from 38 °C, using heating and cooling buttons (rate of change: 0.5 °C/s), until they obtained a level which was felt as barely painful. This procedure was repeated in 4 trials, the first trial was a familiarization trial. The threshold estimate was the average of the last 3 trials. Thereafter, the “pain block” started. Here, 10 painful stimuli (pain threshold +3°C) and 10 non-painful heat stimuli (pain threshold -3°C) were applied in randomized order. The non-painful heat stimuli were applied to have a neutral reference for facial expression analyses. The painful intensity of +3°C above threshold was chosen since this intensity elicits a painful sensation of mild to moderate intensity and thus, the arousal and valence of this painful intensity should be comparable to the disgust pictures we selected. The temperature increased (rate of rise: 4°C/s) from baseline (38°C) to these pre-set temperatures, was kept constant for 5 s (plateau phase) and returned to baseline. Facial as well as subjective responses (ratings) to each stimulus were assessed. 

#### Disgust block

Color pictures of emotional content (800x600 format) were presented for 5 seconds on the computer screen in a randomized order. Pictures were mostly selected from the International Affective Picture System [22]. (Those IAPS pictures, listed by their identification numbers, are as follows: neutral – 219, 1731, 2745.1, 5551, 5720, 5800, 5900,7009, 7041, 7052; pain-disgust – 3101, 3150, 3261, 3400, 7361, 8230, 9405; disgust – 9301, 9320; happy – 1340, 1440, 1441, 1710, 1750, 2091, 2165, 2501, 4625, 7325). Since the IAPS does not include a sufficient number of disgust pictures, 12 pictures were selected from the internet: 3 pain-disgust pictures (content: decubitus, open fracture, suppurated sore) and 8 “pure” disgust pictures (content: moldy toast, rotten teeth, excrements, a woman vomiting, a man vomiting, snot, vomit in a toilet, infected toenail, spitting person). We made sure that the pictures taken from the internet matched the IAPS pictures with regard to arousal and valence and thus, asked 40 individuals in a pilot study to rate the IAPS and non-IAPS pictures. No difference was found between non-IAPS and IAPS pictures (non-IAPS pictures: valence: 3.4 (SD 1.2), arousal: 5.7 (1.3); IAPS pictures: valence: 3.2 (SD 1.1), arousal: 5.9 (SD 1.2); all p-values>0.05). We also included neutral pictures from the IAPS to have a neutral reference for facial expression analyses (as we did with the non-painful heat intensities in the pain block). The picture set also included pictures with happy content, which were only presented to avoid a lowering of mood by only showing negative pictures, but were excluded from further analysis. 

### Assessment of facial responses

Faces of the participants were continuously videotaped throughout both testing blocks using a camcorder (JVC GZ-MG30) mounted on top of the computer screen. Subjects were instructed not to talk during the experimental blocks. A LED behind the subject, visible to the camera, but not to the participant, was lighted concurrently with the (thermal stimuli or pictures) to mark the on- and offset of the stimulation.

Facial expressions were coded from the video recordings using the Facial Action Coding System [9], which is based on anatomical analysis of facial movements and distinguishes 44 different Action Units (AUs) produced by single muscles or combinations of muscles. Two FACS coders identified frequencies and intensities (5-point scale) of all Action Units that occurred during the stimulation (inter-rater reliability =.90 tested in a sub-set (15 %) of the video segments). To segment videos and to enter the FACS codes, we used the Observer Video-Pro (Noldus Information Technology). Time segments of 5 s, beginning just after stimulus onset (in case of the heat stimuli, stimulus onset was defined as start of the 5 second plateau phase), were selected for scoring (the onset of a facial action had to lie in this time window in order to be scored). In total, 20 video segments for thermal stimulation (10 painful and 10 non-painful) and 30 segments of picture presentation (10 neutral, 10 pain-disgust and 10 “pure” disgust) were analyzed per subject. As has been done in preceding studies (especially on facial responses to pain [14,23–25]) we combined those AUs that represent similar facial movements (AU1/2, AU6/7, AU9/10 and AU25/26/27). 

### Self-Report

After stimulus offset, participants were asked to rate their sensations on four separate scales (see Figure 1). Two of these scales were Visual Analogue Scales (VAS; which appeared simultaneously on the screen) which assessed pain and disgust intensity, respectively. Participants were asked to rate both intensities by moving a cursor on the 100 mm VAS scales with the endpoints “no pain” and “extremely strong pain” or “no disgust” and “extremely strong disgust”, respectively. The cursor appeared in random positions to avoid response tendencies due pre-selection of scale ranges. Participants had 16 s to provide both VAS ratings. Following the VAS ratings, participants were asked to rate the valence and arousal of the pain and disgust stimuli using Self Assessment Manikins (SAM [26]) scales that appeared on the computer screen (rating was done by mouse click on the manikins or spaces in-between, resulting into 9 categories). Both SAM scales appeared simultaneously on the screen and participants had 12 seconds to provide their ratings. To familiarize subjects, two practice trials were conducted in each block. 

### Statistical Analyses

#### Ratings of pain and disgust (VAS, SAM)

To investigate whether we succeeded in inducing painful experiences in the pain block and disgust experiences in the disgust block, respectively, we compared VAS ratings between the different types of stimuli (painful heat vs. pain-disgust pictures vs. “pure” disgust pictures) using a multivariate analysis of variance (VAS pain and VAS disgust) with repeated measurement. To investigate whether the different types of stimuli elicited comparable levels of valence and arousal ratings, we again used a multivariate analysis of variance (SAM valence and SAM arousal) with repeated measurement. If a MANOVA revealed significant effects, post-hoc T-Tests (bonferroni-corrected) were conducted for single comparisons.

### Facial responses

To investigate whether facial responses elicited during pain and disgust induction differ from each other we used a two-step approach. In a first step, we analyzed which individual facial actions are elicited during pain and disgust (step 1a) and whether the strength with which these individual facial actions are displayed (frequency and intensity values) differs between the affective states (step 1b). However, given that facial expressions are not only characterized by single facial actions but more importantly by the specific combination of facial actions, we compared in a second step the occurrence of facial action combination during pain and disgust induction. 

#### Step 1a: Which individual facial actions are displayed during pain and disgust experiences, respectively?

First, we wanted to analyze which single Action Units (AUs) are relevant for pain, pain-disgust and “pure disgust” expressions, respectively. For that purpose, we calculated which AUs occurred with a frequency of at least 5% in the segments recorded (this was done separately for pain, pain-disgust and “pure” disgust stimuli; see Table 1 for details). This critical level of 5 % was derived from earlier studies [20,21]. Furthermore, in order for an AU to be classified as being relevant for the respective state, it also had to occur more often during the respective state compared to neutral conditions (neutral pictures or non-painful intensities, respectively). To determine this, effect sizes (Cohen’s *d* [27]) for frequency differences in AUs between pain and non-painful heat, between “pure” disgust and neutral pictures as well as between pain-disgust and neutral pictures were computed. This procedure has been proven to be successful in former studies [20,24.25] to identify AUs that are relevant for specific types of affective states. AUs which showed an effect size d ≥ .05 (medium effect) were selected as pain- or disgust-relevant facial responses (these AUs are displayed bold in Table 1). 

**Table 1:** Frequencies of those facial Action Units (AUs) with a critical frequency of occurrence of more than 5 % during pain and disgust stimulation, respectively.

Action Units	description	pain		„pure“ disgust		pain-disgust	
		percent	*effect size*	percent	*effect size*	percent	*effect size*
AU1/2	brow raiser	14%	d = .47	15%	***d = .56***	15%	*d = .33*
**AU4**	**brow lower**	24%	**d = 0.96**	28%	***d = 1.05***	38%	***d = 1.39***
**AU6/7**	**orbit tightening**	52%	**d = 1.08**	31%	***d= 1.35***	36%	***d = 1.32***
**AU9/10**	**Levator contraction**	8%	**d = .68**	11%	***d = .55***	14%	***d= .55***
AU12	lip corner puller	17%	**d = 0.92**	21%	***d = .58***	6%	*d = -.10*
AU14	Dimpler	11%	d = .45	-	*-*	-	*-*
AU17	chin raiser	7%	d = .40	-	*-*	-	*-*
AU18	lip pucker	7%	d = .33	-	*-*	-	*-*
AU24	lip presser	10%	d = .41	-	*-*	-	*-*
AU25/26/27	mouth opening	24%	**d = 0.73**	7%	*d = .44*	10%	*d = .49*
AU45	Blink	213%	d = .35	104%	*d = -2,52*	89%	*d = -2.90*

Note: Only AUs that occurred in more than 5% of the trials are presented; AUs with at least medium effect size (≥.5) for the difference to the control condition were taken as “relevant” for the respective affective state and marked bold. Printed in bold are those AUs that were relevant in all three affective states Effect sizes indicate the differences in occurrence between affect induction vs. the control conditions (non-painful heat or neutral pictures, respectively) Values are given separately for pain, “pure” disgust and pain-disgust.

#### Step 1b: Are these individual facial actions that encode pain and disgust displayed with similar strength during pain and disgust induction?

In a next step, we analyzed whether those single facial actions that are used to encode pain as well as pain-disgust and “pure” disgust are displayed with similar strength during pain and disgust induction. The strength of each single AU was computed by forming product terms (multiplying the frequency and intensity value of each individual AU). These product terms were then entered into a multivariate analysis of variance with repeated measurement. If the MANOVA revealed significant effects, post-hoc T-Tests (bonferroni-corrected) were conducted for single comparisons.

#### Step 2: Which combinations of single facial actions occur during pain and disgust induction?

In order to compare combinations of facial actions between pain, pain-disgust and “pure disgust” expression, we focused on those AUs that proved to be relevant for all three affective states (see results of step 1a). These were the AUs 4, 6/7 and 9/10, allowing for combinations of 3 elements at maximum. We assessed in which combinations these selected AUs were displayed during pain and during disgust induction and then used chi-square analyses to compare the frequency of facial action combinations between pain, pain-disgust and “pure” disgust expressions.

Statistical analyses were run by means of the statistic software SPSS 21.0. Findings were considered to be statistically significant at p < 0.05. 

## Results

### Ratings of pain and disgust (VAS, SAM)

**Figure 2:**
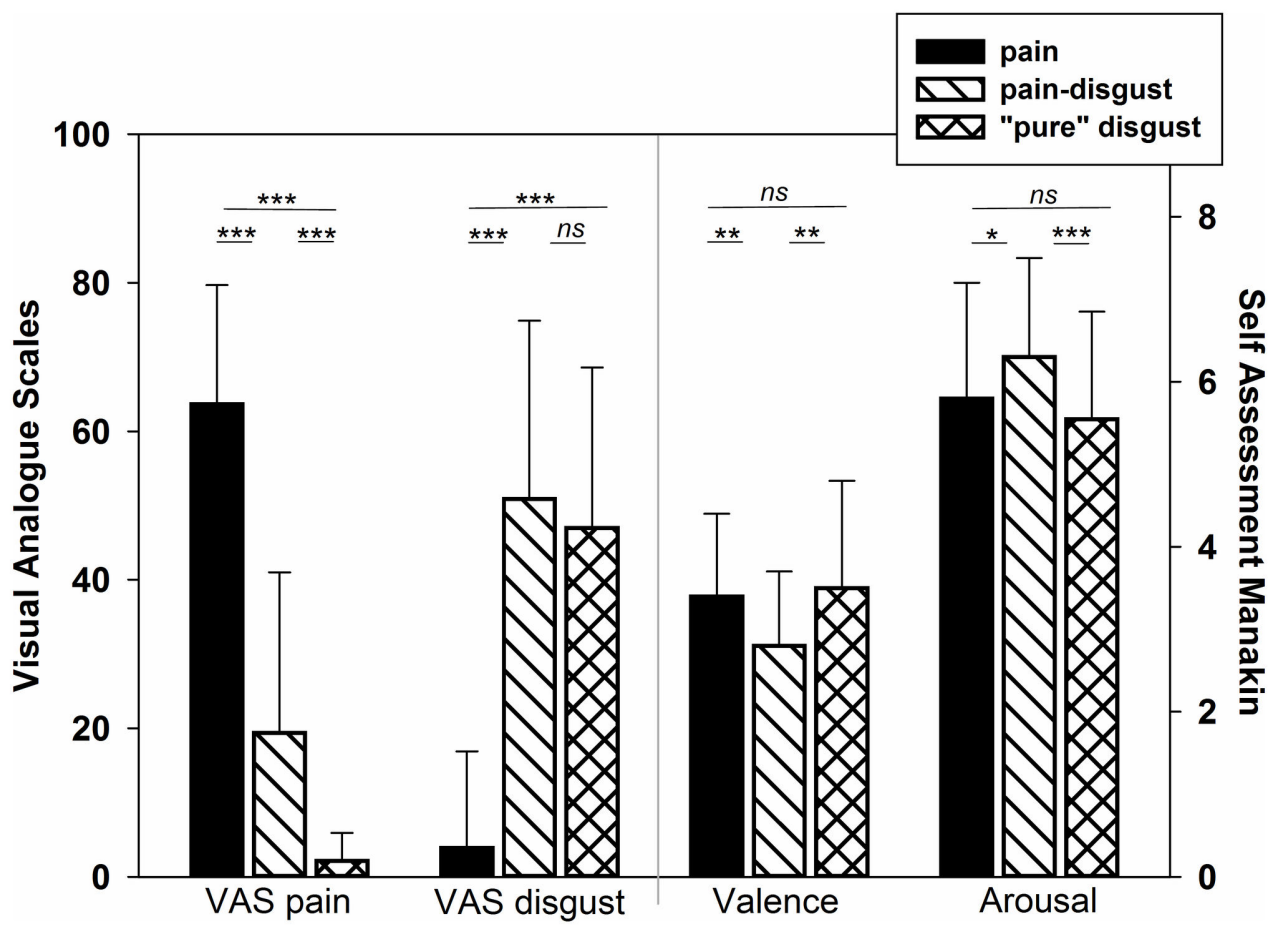
Subjective ratings. Mean values (+SD) of subjective ratings (VAS_pain,disgust_ and SAM_arousal, valence_) during pain, pain-disgust and “pure” disgust induction. Differences of the post-hoc comparisons are displayed (*ns*= not significant, *<.05; **<.01; ***<.001).

VAS: The MANOVA revealed that VAS intensity ratings differed significantly between the different types of stimuli (painful heat vs. pain-disgust pictures vs. “pure” disgust pictures) (F(4,236)=234.00; p<0.001). As the univariate results showed, these differences were significant for the VAS pain ratings (F(2,118)=331,86; p<0.001) as well as for the VAS disgust ratings (F(2,118)=146.47; p<0.001). As expected, pain stimuli were rated to be more painful compared to the disgust pictures (both pain-disgust and “pure” disgust; p-values of the post-hoc comparisons: p<0.001; see also Figure 2). Moreover, the pain-disgust pictures were also rated to be more painful compare to the “pure” disgust pictures (p-value of the post-hoc analysis: p<0.001). With regard to the VAS disgust ratings, participants rated the disgust pictures (both pain-disgust and “pure” disgust) to be more disgusting compared to the pain stimuli (p-values of the post-hoc comparisons: p<0.001; see also Figure 2). VAS disgust ratings of the two picture categories (pain-disgust and “pure” disgust) did not differ significantly (p-value of the post-hoc analysis: p=0.151).

SAM: The MANOVA revealed that the different types of stimuli (painful heat vs. pain-disgust pictures vs. “pure” disgust pictures) elicited significant differences in the SAM ratings (F(4,236)=7.33; p<0.001). As univarate results revealed, both valence (F(2,118)=10.30, p<0.001) as well as arousal ratings (F(2,118)=12.40; p<0.001) changed significantly depending on the types of stimuli. However, as post-hoc comparisons revealed, these differences were only due to pain-disgust pictures being rated to be more arousing and more negative in valence than the pain and “pure” disgust stimuli (all p-values <0.05); whereas pain stimuli and “pure” disgust pictures elicited comparable levels of arousal (p=0.562) and valence ratings (p=0.989) (see also Figure 2). 

*In summary*, our pain and disgust inductions produced similar levels of negative valence and arousal because even though the pain-disgust pictures elicited higher levels of valence and arousal compared to pain and “pure” disgust stimuli, the mean difference in descriptive values between the three types of stimuli was always lower than 1-scale-point on the SAM scales (see Figure 2). In contrast, pain and disgust inductions produced clear differences in pain and disgust VAS ratings and thus, participants appeared to be completely aware of the particular origin of the affective state. Accordingly, we produced conditions that will allow us determining whether facial responses only indicate general valence and arousal of affective states or whether they reflect the specific type of affective state. 

### Facial expression of pain and disgust

#### Step 1a: Which single facial actions are displayed during pain and disgust experiences, respectively?

**Figure 3:**
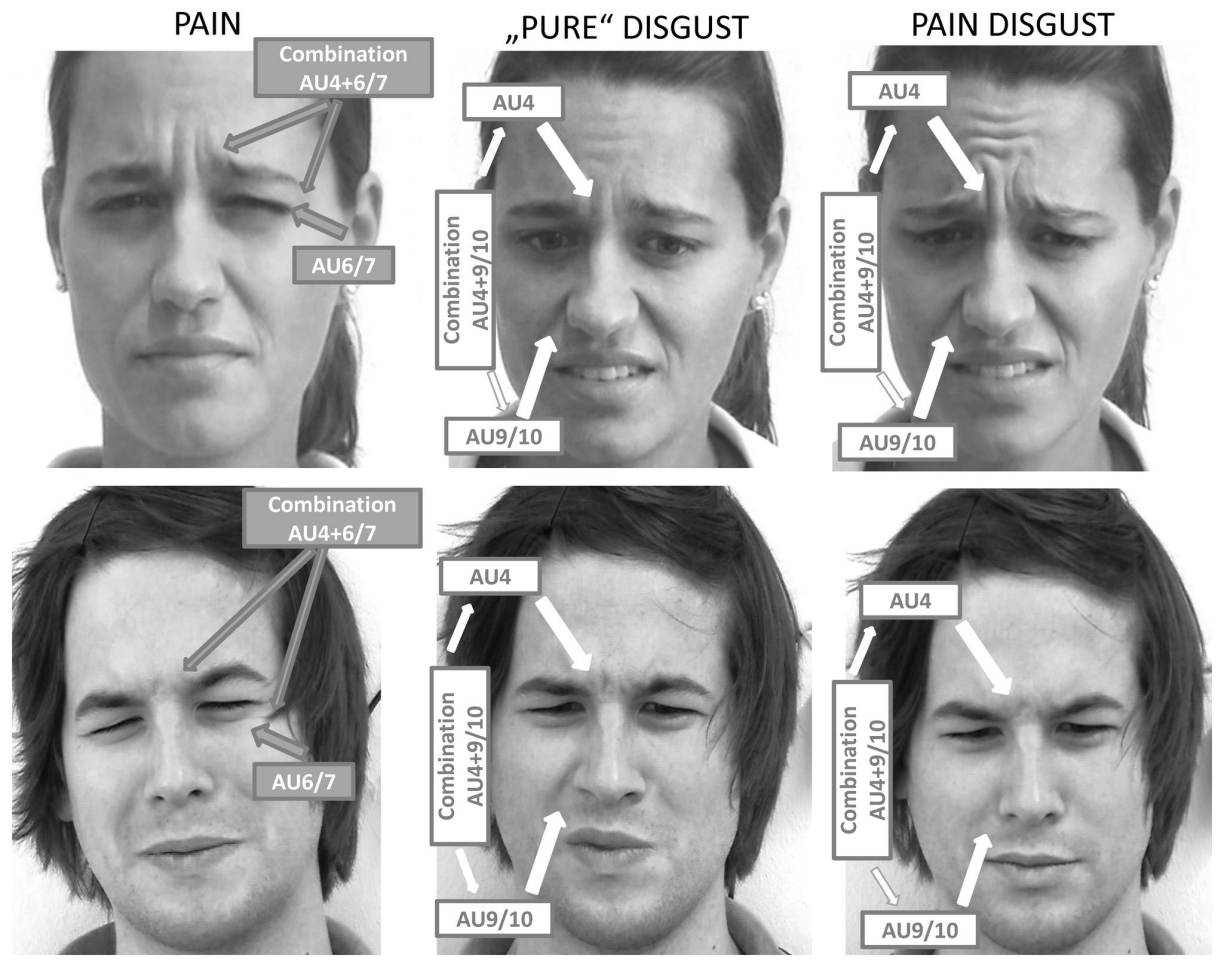
Examples of facial expressions elicited during pain and disgust induction. Facial responses during pain induction (left panel), during pain-disgust induction (middle panel) and during “pure” disgust induction (right panel). The arrows point to the relevant facial actions during pain and disgust, respectively. *The* *subjects* *in* *the* *photograph* *have* *given* *written* *informed* *consent* *as* *outlined* *in* *the* *PLOS* *consent* *form, to* *publication* *of* *their* *photographs*.

Table 1 lists all those AUs that occurred above a critical occurrence level of 5% during pain, pain-disgust and “pure” disgust induction, respectively. As can be seen, pain induction elicited more single facial actions compared to the disgust induction procedures. However, when focusing on those facial actions that proved to be relevant for each of the three types of affective states (medium effect size for the difference to the respective control conditions) there is a great overlap between pain and disgust. As can be seen in Table 1, the facial actions “brow lowering” (AU4), “orbit tightening” (AU6/7) and “levator contraction” (AU9/10) encoded pain as well as the two types of disgust experiences. The only difference between affective states was that pain experience was additionally accompanied by “smiles” (AU12) and “mouth opening” (AU25/26/27) whereas “pure” disgust also led to “brow raising” (AU1/2). Thus, pain and disgust experiences were accompanied by mostly the same facial actions (see also Figure 3 were examples of facial expressions are given).

#### Step 1b: Are these single facial actions that encode pain and disgust displayed with similar strength during pain and disgust experiences?

**Figure 4:**
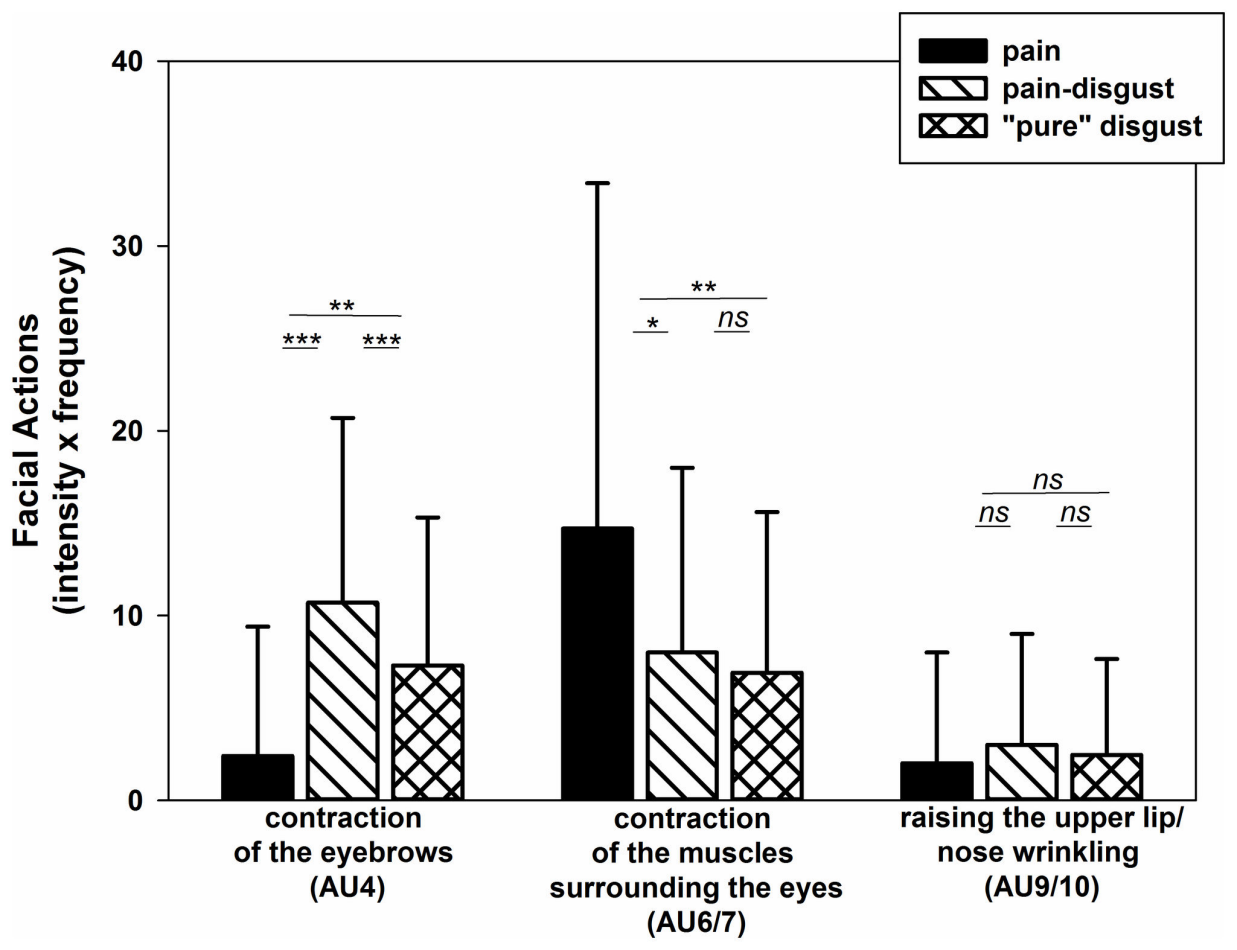
Single facial actions. Mean values (+SD) of those single AUs that were commonly activated during pain, pain-disgust and “pure” disgust” induction. Values are given separately for each affective state. Differences of the post-hoc comparisons are displayed (*ns*= not significant, *<.05; **<.01; ***<.001).

The MANOVA revealed that those single facial actions that encode both pain and disgust are displayed with different strength during pain and disgust induction (F(6,234)=9.39; p<0.001). As the univariate findings showed, AU4 (F(2,118)=19.63; p<0.001) as well as AU6/7 (F(2,118)=9.11; p<0.001) were displayed with different strength depending on the type of affective state, whereas AU9/10 did not differ between affective states (F(2,118)=0.95; p=0.390). When conducting post-hoc analyses for single comparisons we found that AU 4 was displayed less strongly in response to pain induction compared to the two categories of disgust pictures (both p-values <0.010, see also Figure 4). Moreover, AU 4 was displayed more strongly in response to the pain-disgust compared to the “pure” disgust pictures (p<0.001). With regard to AU 6/7, this facial action was displayed most strongly in response to pain induction compared to disgust induction (pain-disgust and “pure” disgust pictures; both p-values <0.050, see also Figure 4) and did not differ between pain-disgust and “pure” disgust (p=0.686) (see also Figure 3 were examples of facial expressions are given).

Given the small but nevertheless significant differences in arousal ratings between affective states (with pain-disgust pictures being rated as being more arousing compared to the other two affective states), we wanted to ensure that our findings on facial responses are indeed not affected by these differences in arousal. To control for this, we divided the group of subjects into those who rated the pain-disgust pictures as more arousing than the other two affective states and those who rated them as equally arousing (median split of the averaged difference scores). We found that the findings on facial responses (as displayed in Figure 4) were not affected by differences in arousal ratings between affective states, given that the same findings were obtained in those individuals who rated the affective states as being differently arousing and in those individuals who rated the affective states as being equally arousing. 

#### Step 2: Which combinations of single facial actions occur during pain and disgust induction?

**Figure 5:**
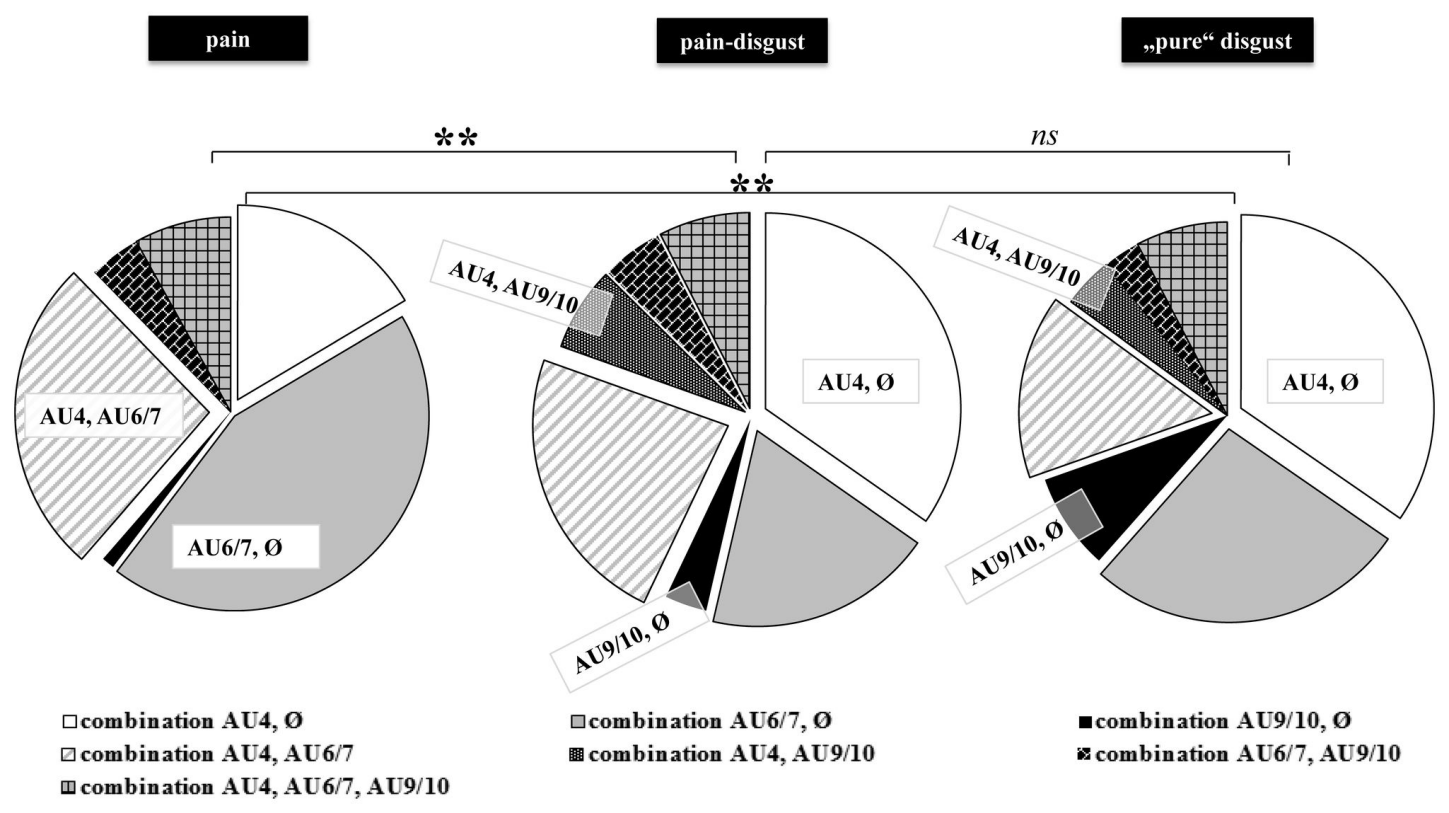
Facial action combinations. Distribution of facial action combinations occurring during pain, pain-disgust and “pure” disgust induction. The sign “∅” indicates that an AU was displayed alone during the 5 seconds of stimulation. Differences between affective states (chi-square test) are displayed (*ns*= not significant, *<.05; **<.01; ***<.001).

As can be seen in Figure 5, we found that in more than half of the segments (both in the pain and in the disgust block) facial expressions were only composed of one single facial action. Especially lowering of the brow (AU4) and orbit tightening (AU6) were often displayed alone (indicated by being combined with “∅” in Figure 5). The most frequent facial action combination was the lowering of the brow together with orbit tightening (combination AU4, AU6/7) both during pain and disgust induction. Despite these similarities, chi-square tests (goodness of fit) revealed significant differences in facial action combinations between pain and “pure” disgust (χ^2^(6)=20.64; p=0.002) as well as between pain and pain-disgust (χ^2^(6)=22.75; p<0.001) induction. In contrast, the two disgust categories (“pure” and pain-disgust) elicited a similar distribution of facial action combinations (χ^2^(6)=5.72; p=0.46). The standardized residuals (stand. res.) - which indicate the importance of each cell to the chi-square value - revealed that brow lowering (AU4) by itself (stand. res. 3.1/3.1), levator contraction (AU9/10) by itself (stand. res. 2.5/1.3) and the combination of these two facial actions (stand. res. 1.6/2.3) occurred markedly more frequently during disgust (“pure” disgust/pain-disgust) compared to pain induction (see Figure 3). In contrast, orbit tightening (AU6/7) by itself (stand. res. 3.3/5.7) and the combination of orbit tightening and brow lowering (AU4) (stand. res. 2.8 for the difference to “pure” disgust) occurred markedly more frequent during pain (see Figure 3) compared to “pure” disgust/pain-disgust induction. 

*In summary*, pain and disgust experiences seem to elicit the same single facial actions. However, the strength, with which these single facial actions are displayed during pain and disgust differs significantly, even when controlling for differences in arousal ratings. Moreover, when considering combinations of single facial actions, we also found clear differences between pain and disgust. Whereas brow lowering (AU4), levator contraction (AU9/10) and the combination of these two facial responses are more pronounced during disgust, pain seems to be encoded by a more pronounced orbit tightening (AU6/7) (often in combination with brow lowering (AU4)) (see Figure 3). 

## Discussion

The aim of our study was to investigate whether facial displays occurring during the experience of pain and of disgust overlap substantially and indistinguishably or whether they are distinct enough to communicate different states. Pain and disgust were selected for this comparison because both are elicited by actual or potential harm to the body, are characterized by strong feelings of unpleasantness, result into defensive behavior and facial expressions of both states are often confused by observers [4,5]. Facial responses to pain and disgust are ideal models to test the distinctiveness of facial expressions because of their expressive similarity, which guarantees - in case of proven differences in facial encoding - differential encoding also for most of the other emotional states. It has to be kept in mind that the strong similarity between the two states exists, although the subjective experience clearly reflects the specific type of threat and allows for differential self-reports.

Our main findings were that nearly the same single facial actions were elicited during the experience of pain and disgust. However, these facial actions were displayed with different strength and were differently combined depending on whether pain or disgust was experienced. We will discuss these findings in more detail below.

As stated above, we found that nearly the same single facial muscle movements were elicited during the experience of pain and disgust. More precisely, the contraction of the eyebrows (orbicularis oculi muscle; AU 6/7), contraction of muscles surrounding the eyes (corrugator muscle; AU4) and lifting the upper lip (levator muscle; AU9/10) were observed both during pain and disgust induction. This overlap in facial actions is well in line with previous findings which have also reported similar facial actions in response to pain [13–15] and disgust induction [10–12] (although this is the first study to directly compare facial responses to pain and disgust in one sample). Given that we induced both pain and disgust in one sample we were also able to directly compare the strength to which each of these facial actions was displayed during pain and disgust experiences. (For note, the ratings of valence and arousal were very similar during disgust and pain.). We found that although contraction of the muscles surrounding the eyes (AU6/7) occurred both during pain and disgust, this facial action was more relevant for pain than it was for disgust (see also examples displayed in Figure 3). This is in line with previous assumptions that the orbicularis oculi activation is the most prominent facial response to pain [13]. In contrast, the contraction of the eyebrows occurred more strongly during disgust compared to pain induction. Moreover, facial expressions of pain and disgust could be even better differentiated when considering how these single facial actions were combined. The most frequent facial response that can be observed when an individual is experiencing pain seems to be the contraction of the muscles surrounding the eyes (AU6/7), either by itself or in combination with contraction of the eyebrows (AU4), which accounted for nearly 2/3 of all facial responses to pain in the present study. Facial expressions of disgust, on the other hand, seem to be more variable than pain expressions. Most often contraction of the eyebrows (AU4) is displayed by itself. In contrast to pain, disgust is also more often encoded by raising the upper lip/wrinkling the nose (AU9/10) by itself as well as by the combination of eyebrow contraction and upper lip raise (see also Figure 3 for examples of facial expressions). Consequently, although the same facial actions are used to facially encode pain and disgust, these facial actions are differently combined and displayed with different strength during pain and disgust experiences, thus, suggesting that the facial displays can indeed signal categorically distinct states although similar muscles are involved. 

The literature on decoding of affective states by observers has introduced two perspectives a discrete-category and a dimensional view [2,28,29], which are worthwhile being considered here; although our experiment dealt with the encoding of facial displays by senders and not with the decoding by receivers. According to the dimensional view [30,31] facial expressions mainly convey values on the dimensions of valence and arousal. Given that pain and disgust elicit very similar valence and arousal ratings one would expect – based on the dimensional view – also very similar facial responses to pain and disgust. In accordance with this expectation (namely that facial responses mainly convey valence and arousal information), we indeed found a great overlap of single facial actions that encode both pain and disgust. In other words, the type of single facial actions being displayed might convey mainly information on valence and arousal to the observer. However, we also found that despite the great overlap of single facial actions, facial responses to pain and disgust are also able to signal categorically discrete states. This is in line with the discrete-category view hypothesized that reading the facial expression of emotions results into clearly differential classifications because each emotion has its specific facial readout [2]. Our data support such a hypothesis with regard to the affective states pain and disgust. When we took into consideration not only the type of single facial actions but the strength to which they are displayed as well as how they are combined, facial expressions of pain and disgust were clearly distinct. Thus, the types of single facial actions being elicited seem to convey information on valence and arousal (dimensional view) whereas the strength and the combination in which they are displayed seem to form the basis for communicating differentially emotional states, allowing for their identification by an observer (discrete-category view). Given that the differential classification of disgust and pain is amongst the most difficult ones, our data also corroborate a more general conclusion. 

We used two different picture categories to induce disgust, namely pictures showing pain-related content (e.g. mutilation) as well as pictures without any pain-related content (e.g. body waste products) to investigate whether the previously reported overlap between facial responses to pain and disgust might be simply due to facial expressions of disgust having been elicited by pain-confounded stimuli [18,19]. We found no clear differences between facial expressions in response to pain-disgust and “pure” disgust pictures. Moreover, facial responses to the pain-disgust pictures were not more similar to pain than the “pure” disgust responses. Thus, our data clearly suggest, that this confound of disgust with pain-related content is not responsible for the great overlap in facial actions elicited during the experience of pain and disgust. 

As a limitation of the present study, it has to be mentioned that the methods used to induce pain and disgust were quite different. Whereas for the pain induction procedure, a physical stimulus was used to directly induce the affective state “pain”, we induced the affective state “disgust” by use of picture stimuli, which are only icons of the actual threat to the body. The reason for using pictures to induce disgust was that we wanted to include both “pure” disgust and pain-confounded disgust stimuli, which cannot be all made available as physical stimuli. As explained above, we did this to control - for the first time - for this confound of pain and disgust contents in some of the IAPS designated disgust pictures. 

In summary, investigating facial and subjective responses to pain and disgust induction in one sample of participants revealed that the single facial actions elicited during the two states are rather similar than different. This similarity is paralleled by the similarity of the valence and arousal ratings. However, when considering the strength with which each of these single facial actions are displayed and how these single facial actions are combined during the experience of pain and disgust, significant differences occurred. Consequently, facial expressions of pain and disgust seem distinct enough to also encode the specific type of the threat to the body. Thus, facial expressions seem to be able to signal both, the general valence as well as arousal and the specific threat to the body. This implies that the differential decoding of these two states by an observer is possible without additional verbal or contextual information. This is of special interest for clinical practice, given that raising awareness in observers about these distinct differences could help to improve the detection of pain in patients who are not able to provide a self-report of pain (e.g. patients with dementia). Moreover, in future studies the question should be answered how the decoding of the facial activity during pain and disgust is affected when systematically varying the strength and combination of single facial actions (e.g. by using animated avatar facial expressions). The findings of these studies may confirm that the features we found in the present study are indeed crucial for the identification of pain and disgust by observers. 

## References

[1] Fridlund JA (1994) Human facial expression: An ecolutionary view. San Diego: Academic Press.

[2] Ekman P (1992) An argument for basic emotions. Cognition and Emotion 6: 169–200. doi:10.1080/02699939208411068.

[3] Williams ACC (2002) Facial expression of pain: An evolutionary account. Behav Brain Sci 25: 439-488. PubMed: 12879700. 12879700 10.1017/s0140525x02000080

[4] Simon D, Craig KD, Gosselin F, Belin P, Rainville P (2008) Recognition and discrimination of prototypical dynamic expressions of pain and emotions. Pain 135: 55-64. doi:10.1016/j.pain.2007.05.008. PubMed: 17583430. 17583430

[5] Kappesser J, Williams AC (2002) Pain and negative emotions in the face: judgements by health care professionals. Pain 99: 197–206. doi:10.1016/S0304-3959(02)00101-X. PubMed: 12237197. 12237197

[6] Curtis V, Aunger R, Rabie T (2004) Evidence that disgust evolved to protect from risk of disease. Proceedings of the Royal Society of London, Series B 271: S131–S133. doi:10.1098/rsbl.2003.0144. PubMed: 15252963. 15252963 PMC1810028

[7] Curtis V, Biran A (2001) Dirt, disgust, and disease: Is hygiene in our genes? Perspect Biol Med 44: 17–31. doi:10.1353/pbm.2001.0001. PubMed: 11253302. 11253302

[8] Merskey H, Bogduk N (2002) Classification of chronic pain, descriptions of chronic pain syndromes and definitions of pain terms. IASP Press.

[9] Ekman P, Friesen WV (1978) The Facial Action Coding System (FACS): A technique for the measurement of facial action. Palo Alto. CA: Consulting Psychologists Press.

[10] Ekman P, Friesen WV (1975) Unmasking the face: A guide to recognizing emotions from facial clues. Englewood Cliffs, NJ: Prentice Hall.

[11] Smith C, Scott H (1997) A componential approach to the meaning of facial expressions. In Russel J Fernandez-Dols J, The psychology of facial expression. Cambridge: Cambridge University Press.

[12] Rozin P, Lowery L, Ebert R (1994) Varieties of disgust faces and the structure of disgust. J Pers Soc Psychol 66: 870–881. doi:10.1037/0022-3514.66.5.870. PubMed: 8014832. 8014832

[13] Craig KD, Prkachin KM, Grunau RVE (2011) The facial expression of pain. In Turk DC Melzack R Handbook of pain assessment, 3rd ed. New York: Guilford pp. 117-133.

[14] Prkachin KM (1992) The consistency of facial expressions of pain: a comparison across modalities. Pain 51: 297-306. doi:10.1016/0304-3959(92)90213-U. PubMed: 1491857. 1491857

[15] Prkachin KM, Solomon PE (2008) The structure, reliability and validity of pain expression: evidence from patients with shoulder pain. Pain 139: 267-274. doi:10.1016/j.pain.2008.04.010. PubMed: 18502049. 18502049

[16] Hale C, Hadjistavropoulos T (1997) Emotional components of pain. Pain Research and Management 2: 217-225.

[17] Keltner D, Buswell BN (1996) Evidence for the distinctness of embarrasement, shame and guilt: a study of recalled antecedents and facial expressions of emotion. Cognition Emotion 10: 155-171. doi:10.1080/026999396380312.

[18] Wolf K, Mass R, Ingenbleek T, Kiefer F, Naber D et al. (2005) The facial pattern of disgust, appetence, excited joy and relaxed joy: an improved facial EMG study. Scand J Psychol 46: 403-409. doi:10.1111/j.1467-9450.2005.00471.x. PubMed: 16179022. 16179022

[19] Rohrmann S, Hopp H, Schienle A, Hodapp V (2009) Emotion regulation, disgust sensitivity, and psychophysiological responses to disgust-inducing films. Anxiety, Stress and Coping 22: 215-236. doi:10.1080/10615800802016591. 19259873

[20] Kunz M, Chen JI, Lautenbacher S, Vachon-Presseau E, Rainville P (2011) Cerebral regulation of facial expressions of pain. J Neurosci 31: 8730-8738. doi:10.1523/JNEUROSCI.0217-11.2011. PubMed: 21677157. 21677157 PMC6622930

[21] Kunz M, Lautenbacher S, LeBlanc N, Rainville P (2012) Are both the sensory and the affective dimensions of pain encoded in the face? Pain 153: 350-358. doi:10.1016/j.pain.2011.10.027. PubMed: 22112930. 22112930

[22] Lang PJ, Bradley MD, Cuthbert B (1995) International Affective Picture System. Gainsville: Center for Research in Psychophysiology, University of Florida.

[23] Kunz M, Mylius V, Schepelmann K, Lautenbacher S (2004) On the relationship between self-report and facial expression of pain. J Pain 5: 368-376. doi:10.1016/j.jpain.2004.06.002. PubMed: 15501194. 15501194

[24] Kunz M, Mylius V, Schepelmann K, Lautenbacher S (2008) Impact of age on the facial expression of pain. J Psychosom Res 64: 311-318. PubMed: 18291247. 18291247 10.1016/j.jpsychores.2007.09.010

[25] Kunz M, Chatelle C, Lautenbacher S, Rainville P (2008) The relation between catastrophizing and facial responsiveness to pain. Pain 140: 127-134. doi:10.1016/j.pain.2008.07.019. PubMed: 18783885. 18783885

[26] Bradley MM, Lang PJ (1994) Measuring emotion: The self assessment manikin and the semantic differential. J Behav Ther Exp Psychiatry 25: 49-59. doi:10.1016/0005-7916(94)90063-9. PubMed: 7962581. 7962581

[27] Cohen J (1977) Statistical power analysis for the behavioral sciences. New York: Academic Press.

[28] Aviezer H, Hassin R, Ryan J, Grady C, Susskind J et al. (2008) Angry, disgusted, or afraid? Studies on the Malleability of Emotion Peception. Psycological Science 19: 724-732. 10.1111/j.1467-9280.2008.02148.x 18727789

[29] Carroll JM, Russell JA (1996) Do facial expressions signal specific emotions? Judging emotion from the face in context. J Pers Soc Psychol 70: 205–218. doi:10.1037/0022-3514.70.2.205. PubMed: 8636880. 8636880

[30] Russell JA (1980) The circumplex model of affect. Journal of Personality and Social Psychology 39: 1161-1178. doi:10.1037/h0077714. 10948981

[31] Russell JA (1997) Reading emotions from and into faces: Resurrecting a dimensional contextual perspective. In Russell JA Fernandez-Dols JM, The psychology of facial expressions. New York: Cambridge University Press (pp. 295–320).

